# Therapeutic Applications of Mesenchymal Stem Cells in Idiopathic Pulmonary Fibrosis

**DOI:** 10.3389/fcell.2021.639657

**Published:** 2021-03-09

**Authors:** Shengnan Yang, Peipei Liu, Yale Jiang, Zai Wang, Huaping Dai, Chen Wang

**Affiliations:** ^1^Department of Pulmonary and Critical Care Medicine, Center of Respiratory Medicine, China-Japan Friendship Hospital, Beijing, China; ^2^National Center for Respiratory Medicine, Beijing, China; ^3^Institute of Respiratory Medicine, Chinese Academy of Medical Sciences, Beijing, China; ^4^National Clinical Research Center for Respiratory Diseases, Beijing, China; ^5^WHO Collaborating Centre for Tobacco Cessation and Respiratory Diseases Prevention, Beijing, China; ^6^Harbin Medical University, Harbin, China; ^7^Graduate School of Peking Union Medical College, Chinese Academy of Medical Sciences and Peking Union Medical College, Beijing, China; ^8^School of Medicine, Tsinghua University, Beijing, China; ^9^Institute of Clinical Medical Sciences, China-Japan Friendship Hospital, Beijing, China; ^10^Chinese Academy of Medical Sciences and Peking Union Medical College, Beijing, China

**Keywords:** mesenchymal stem cells, idiopathic pulmonary fibrosis, stem cell transplantation, interstitial lung disease, interstitial fibrosis

## Abstract

Idiopathic pulmonary fibrosis (IPF) is an interstitial disease of unknown etiology characterized by progressive pulmonary fibrosis. Pirfenidone and nintedanib are the only drugs that can prolong the time to disease progression, slow down the decline in lung function, and prolong survival. However, they do not offer a cure and are associated with tolerability issues. The pluripotency of mesenchymal stem cells (MSCs) and their ability to regulate immunity, inhibit inflammation, and promote epithelial tissue repair highlight the promise of MSC therapy for treating interstitial lung disease. However, optimal protocols are lacking for multi-parameter selection in MSC therapy. This review summarizes preclinical studies on MSC transplantation for the treatment of interstitial lung disease and clinical studies with known results. An analysis of relevant factors for the optimization of treatment plans is presented, including MSCs with different sources, administration routes and timing, dosages, frequencies, and pretreatments with MSCs. This review proposes an optimized plan for guiding the design of future clinical research to identify therapeutic options for this complex disease.

## Idiopathic Pulmonary Fibrosis and Mesenchymal Stem Cell Therapy

Idiopathic pulmonary fibrosis (IPF) is a chronic and irreversible interstitial lung disease characterized by progressive pulmonary fibrosis (Raghu et al., 2018). In high-resolution CT scans, IPF presents with typical imaging features of interstitial pneumonia (UIP) as reticular and honeycomb opacities, mainly distributed in subpleural and basal regions, accompanied by traction bronchiectasis. Pathologically, UIP lung tissue exhibits heterogeneous interstitial fibrosis, structural deformation, and fibroblast foci (Martinez and Flaherty, 2017). Disease prognosis is poor, with an average life expectancy of 3–5 years after diagnosis if untreated (Lederer and Martinez, 2018; Raghu et al., 2018) and a mortality rate exceeding that of many malignancies (Tzouvelekis et al., 2015; Karampitsakos et al., 2017).

Over the past two decades, significant progress has been made in understanding the pathogenesis of IPF. In genetically susceptible aging individuals, recurrent environmental and/or repetitive alveolar epithelial micro-injury has been recognized as the trigger of disordered repair. Dysfunction of the alveolar epithelium is considered a key step in the initiation of IPF disease. Many factors that might underlie the dysfunction of epithelial cells in IPF have been proposed, including telomere shortening, aberrant mitochondrial bioenergetics, and increased endoplasmic reticulum stress induced by the unfolded protein response (UPR) (Lawson et al., 2011; Naikawadi et al., 2016). A relevant consequence of this is the production of profibrotic mediators, such as transforming growth factor-beta 1 (TGF-β1), platelet-derived growth factor (PDGF); chemokine C-C motif ligand 2 (CCL2); and C-X-C motif chemokine 12 (CXCL12) (Sgalla et al., 2018). TGF-β1 is a powerful pro-fibrotic mediator that promotes epithelial cell apoptosis, epithelial-mesenchymal transition, and the production of other profibrotic mediators, such as vascular endothelial growth factor (VEGF), connective tissue growth factor (CTGF), and other pro-angiogenic mediators, forming a positive feedback loop (Grimminger et al., 2015). Dysregulated epithelial-fibroblast cross-talk promotes myofibroblast accumulation and excessive matrix deposition, which lead to continuous tissue remodeling and structural deformations (Barratt et al., 2018; Ballester et al., 2019). In addition, immune dysfunction is also one of the drivers of IPF (Shenderov et al., 2021). CD11b^+^F4/80^+^Ly6C^+^ inflammatory macrophages can recruit and activate Th2 cells in lung tissue, and an imbalance in the Th1/Th2 immune response is considered to contribute to IPF pathogenesis (Morimoto et al., 2018). Macrophages are also an effective source of fibrotic cytokines (such as TGF-β1 and PDGF), chemokines, and proteases (Gordon et al., 2014) and promote collagen synthesis by upregulating arginine metabolism (Wang et al., 2019). The pathogenesis of IPF is complex, and the disease course is difficult to predict. With the lack of effective treatments, patients inevitably progress to end-stage respiratory failure and have a higher risk of lung cancer (Caminati et al., 2019). However, the use of antifibrotic drugs such as pirfenidone (Costabel et al., 2017) and nintedanib (Richeldi et al., 2018) has been shown to slow down the fibrotic progression and likely improve progression-free survival. However, both drugs have noticeable gastrointestinal side effects (Bargagli et al., 2019). Although lung transplantation might improve the quality of life and survival, various challenges exist, such as a shortage of donor organs, immune rejection, and surgical complications (George et al., 2019). Accordingly, novel treatment strategies that are safe, effective, and convenient need to be urgently developed.

**FIGURE 1:**
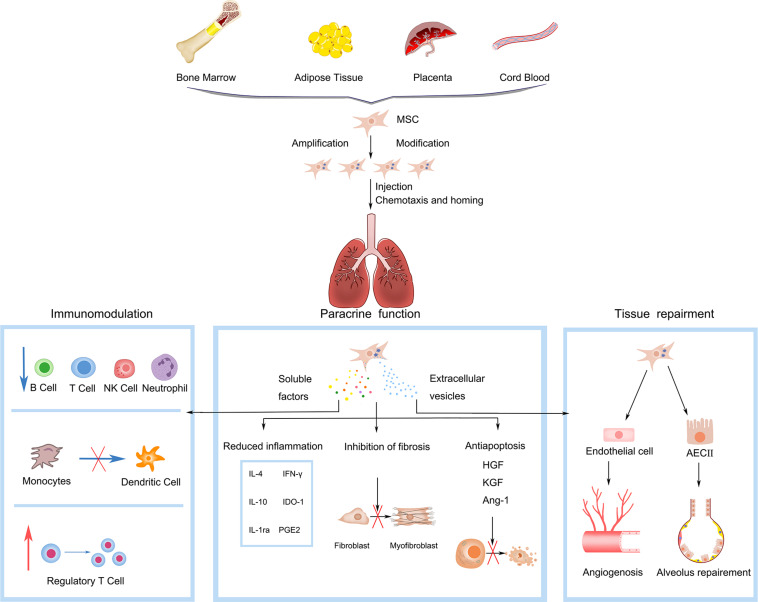
Mesenchymal stem cells (MSCs) gain capability of chemotaxis and homing to damaged lung by *in vitro* amplification and genetic engineering modification. Functions of MSCs in pulmonary fibrosis include: (1) Immunoregulation, interacting with multiple immune cells, such as T lymphocyte cell (T cell), natural killer (NK) cell, dendritic cell (DC), and B lymphocyte cell (B cell); blue arrows refer to inhibition, red arrows refer to promotion. (2) Paracrine function, secreting soluble factors and extracellular vesicles with the functions of reducing inflammation (IL-10, IL-4, IL-1ra, IFN-γ, PGE2, IDO-1), anti-apoptotic (Ang-1, HGF, KGF) and anti-fibrosis. (3) Tissue repairment, interacting with endothelial and epithelial cells to promote angiogenesis and alveolar repairment. IL-10, interleukin-10; IL-4, interleukin-4; IL-1ra, IL-1 receptor antagonist; IFN-γ, interferon-γ; PGE2, prostaglandin E2; IDO-1, indolamine 2,3-dioxygenase-1; Ang-1, angiogenin-1; HGF, hepatocyte growth factor; KGF, keratinocyte growth factor.

Mesenchymal stem cells (MSCs) reside in the perivascular niche *in vivo*. Upon local injury, they can be activated and recruited to the site of injury. By secreting bioactive molecules and regulating local immune response, they can establish a regeneration-promoting microenvironment (Caplan and Correa, 2011). In 1995, Lazarus et al. (1995) first tested MSCs as cellular drug preparations in human subjects. These have since emerged as the most extensively studied cells for experimental cell therapy globally. MSCs have the advantages of originating from a wide range of sources, easy availability, extensive proliferation properties, fewer ethical disputes, and low immunogenicity (Najar et al., 2019). After *in vitro* expansion and allogenic infusion, MSCs can still be recruited to sites of injury, promote epithelial tissue repair, and have powerful immunomodulatory properties such as inhibiting inflammation. These properties make MSCs ideal candidates for tissue engineering, regenerative medicine, and cell-based therapy for IPF (Lu and El-Hashash, 2019). It was initially thought that the benefits of MSC therapy were due to the replacement of damaged cells by these cells during tissue repair. However, subsequent experimental data revealed that the replacement of damaged cells was not the primary mechanism for MSC efficacy. Emerging evidence suggests that these cells exert their tissue repair-promoting and immunomodulatory effects through direct intercellular interactions or the secretion of bioactive products, termed the secretome, which comprises a series of bioactive molecules and extracellular vesicles (EVs). For their tissue repair-promoting effect, MSCs secrete various growth factors, including keratinocyte growth factor (FGF), hepatocyte growth factor (HGF), epidermal growth factor (EGF), and angiogenesis factors, which promote re-epithelialization and angiogenesis (Cahill et al., 2016; Lan et al., 2017; Li et al., 2017d). In addition, by direct mitochondrial transfer via connexin-mediated intercellular channels or EVs from MSCs to the damaged cells, MSCs can restore the ATP storage in recipient cells and repair cell functions (Morrison et al., 2017; Paliwal et al., 2018). For their immunomodulatory role, MSCs express a set of injury and molecular pathogen receptors, such as Toll-like receptors (Shirjang et al., 2017), and release a series of cytokines and chemokines, such as IL-1 receptor antagonist (IL-1RA) (Harrell et al., 2020) and soluble TNF receptor 1 (sTNFR1) (Ding et al., 2019), which have anti-inflammatory effects. Intercellular contact molecules or MSC-secreted soluble factors regulate the adaptive and innate immune system by inhibiting the maturation of T cells and dendritic cells, reducing B cell activation and proliferation, and inhibiting the cytotoxicity of natural killer cells (Ni et al., 2018; de Castro et al., 2019; He et al., 2020). MSCs modulate macrophage phenotypes by reducing the proportion of the pro-fibrotic cell phenotype (M2) and exerting anti-fibrotic effects (Willis et al., 2018; Luo et al., 2019). Furthermore, they directly counteract the fibrotic process by modulating the ratio of metalloproteinases/metalloproteinase tissue inhibitors, thereby reducing the content of collagen fibers and inhibiting lung remodeling (Xu et al., 2017; Chu et al., 2019). A summary of the therapeutic properties and mechanisms of MSCs in pulmonary fibrosis is shown in Figure 1.

## Multifactor Selection for the Treatment of IPF With MSCs

Over the past 10 years, the therapeutic potential of MSCs for the restoration of injured lungs has received considerable interest. Knowledge of the mechanistic involvement of MSCs in pulmonary fibrosis is mainly derived from preclinical rodent models and *in vitro* analysis of human MSCs. Typical experimental protocols include the isolation of MSCs, plastic adherent cultures, harvesting of logarithmic growth phase cells, and adoptive transplantation in animal models of pulmonary fibrosis (Zhang et al., 2019; Gad et al., 2020; Periera-Simon et al., 2021). Although MSCs can reduce fibrosis, the various experimental variables obfuscate direct comparisons between different experiments. The tissue source, type of cells, delivery mode, dose, speed, and frequency of infusion are important parameters that affect the therapeutic effects of cell therapy, but these are challenging to test in large-scale human studies. Further preclinical experimental data on these parameters is necessary to improve the design of clinical intervention trials and ensure the safety and evidence-based nature of MSC treatments.

### Cell Type

Even slight changes in cell type, cell source, and pretreatment methods can affect the efficacy of the final product. The design of MSC-based products must consider these key parameters for appropriate interpretation of results and to provide useful guidance for clinical trial design (Waszak et al., 2012).

#### Comparison of MSCs From Different Tissues

MSCs have been already isolated from a variety of adult and perinatal tissues, including but not limited to bone marrow, adipose tissue, dental pulp, menstrual blood, placenta, amnion, umbilical cord blood, and Wharton’s jelly (Brown et al., 2019; Chen et al., 2020). MSCs from different sources exhibit different immunomodulatory abilities, *in vitro* proliferation properties and *in vivo* therapeutic functions. Among them, bone marrow-derived MSCs (BM-MSCs) were the first type of isolated MSCs and the most commonly used cell type for lung diseases (Antunes et al., 2014b), which may have a better immunomodulatory ability than MSCs from other sources. Compared to adipose-derived MSCs (AD-MSCs), BM-MSCs have been demonstrated to more effectively regulate the phenotypic transition of macrophages in various lung injury models (Antunes et al., 2014a; Kitoko et al., 2018). By co-culturing them with spleen mononuclear cells, BM-MSC could suppress the expression of CD4 and CD8, whereas AD-MSCs could only inhibit the expression of CD4 (El-Sayed et al., 2019). When the immunomodulatory activity of MSCs was assessed based on their ability to suppress the phytohemagglutinin-induced proliferation of peripheral blood mononuclear cells, BM-MSCs showed significantly higher activity than AD-MSCs and Wharton’s jelly MSCs (WJ-MSCs) (Petrenko et al., 2020). Therefore, BM-MSCs might be the best cell source for immune regulation.

The proliferation rate of MSCs also varies according to the tissue source. Recent studies have shown that average population doubling time (PDT) of WJ-MSCs is shorter than 24 h and stable for at least five passages (Petrenko et al., 2020). Choudhery et al. (2013) showed that PDT was shorter for umbilical cord MSCs (UC-MSCs) than for AD-MSCs. Another study demonstrated that compared to that with BM-MSCs, WJ-MSCs have a higher proliferation capacity (Batsali et al., 2017). That study also revealed differences in proliferation activity between BM-MSCs and AD-MSCs. From passage 2, BM-MSCs showed a longer PDT and entered senescence (Petrenko et al., 2020). Similar observations were reported by Kern et al. (2006), showing that BM-MSCs possessed the lowest proliferation capacity compared to that with AD-MSCs or UC-MSCs. Taken together, comparing UC-, AD-, and BM-MSCs, UC-MSCs show a highest proliferation activity and BM-MSCs show the lowest. In addition, the painful invasive isolation procedure for BM-MSCs has called for the identification of alternative sources for MSCs, and UC-MSCs, which are treated as biological waste and usually discarded after birth, could be one of these choices.

In terms of *in vivo* therapeutic functions, especially for treating lung fibrosis, MSCs from different sources have also shown different effects. Pereira-Simon et al. studied the effect of various sources of MSCs in treating bleomycin (BLM)-induced lung fibrosis in aging mice, including AD- and WJ-MSCs, and MSCs derived from the chorionic membrane (CSC) and chorionic villi. All sources decreased Aschroft score at day 10 post-treatment; further, all sources except CSC decreased hydroxyproline levels and α_*v*_-*integrin* and *TNF*-α mRNA levels. Meanwhile, only AD- and WJ-MSCs reduced AKT and MMP-2 activation, whereas Cav-1 was increased by AD-MSCs. Only AD-MSCs restored BLM-induced miR dysregulation of miR-29 and miR-199. These results showed that sources of MSCs might differ in their repair functions and underlying mechanisms (Periera-Simon et al., 2021). The latest single-cell RNA sequencing study has identified very significant transcriptional overlap between WJ- and AD-MSCs, thus potentially explaining some of these findings (Sun et al., 2020). MSCs from different tissues applied in animal models of pulmonary fibrosis are demonstrated in Table 1.

**TABLE 1:** MSCs from different tissues applied in animal models of pulmonary fibrosis.

Source	PF model	Dose	Delivey	Time of cell transplantation	Repeated application	Efficacy results	References
BM	Rat, amiodarone-induced	3 × 10^6^	IV	3 months a.t.	No	↓ CC16, COL1A1, CTGF ↑ KGF levels	Abdel Halim et al., 2020
BM	Rat, BLM-induced	1 × 10^6^	IV	14 days a.t.	No	↓ TGF-β/SMAD3 expression ↑ TNF-α and IL-6	Gad et al., 2020
BM	Rat, silica-induced	2 × 10^6^	IV	28 days a.t.	No	↓ Wnt/β-catenin signaling and EMT	Zhang et al., 2018
BM	Mice, BLM-induced	5 × 10^5^	IV	2 days a.t.	No	↓ T-cell infiltration ↑ TNF-α, IFN-γ and IP-10	Ni et al., 2018
AD	Rat, silicosis-induced	5 × 10^5^	IV	24 h a.t.	No	↓ Caspase-3 protein ↑ Bcl-2/Bax ratio ↑ Anti-inflammatory factors	Chen et al., 2018
AD	Rat, radiation-induced	5 × 10^6^	IV	2 h; 7 days; 2 h+ 7 days a.t.	Yes	↓ EMT, TNF-α, IL-1 and IL-6 ↑ IL-10 and IL-2	Zhang et al., 2019
AD	Mice, BLM-induced	5 × 10^5^	IV	24 h a.t.	No	↓ miR-199,caveolin-1 and phosphorylation of protein kinase B	Rubio et al., 2018
AD	Mice, BLM-induced	40 × 10^6^/kg	IV	3, 6 and 9 days a.t.	Yes	↓ Profibrotic and pro-inflammatory gene transcripts	Reddy et al., 2016
WJ	Rat, BLM-induced	5 × 10^6^ 2.5 × 10^7^	IT	21 days a.t.	No	↓ EMT ↑ MMP-9 and TLR-4	Chu et al., 2019
Placental	Mice, BLM-induced	1 × 10^5^	IV	3 days a.t.	No	↓ HYP, TGF-β, TNF-α, IL-1β; ↓ MyD88/TGF-β signaling axis	Li et al., 2017b
Amniotic	Rat, paraquat-induced	2 × 10^6^	IV	6 h a.t.	No	↓ Inflammatory CD3 + T cell infiltration; ↓ TNF-α, IL-6, TGF-β1 and lactic acid	He et al., 2020
Menstrual blood	Mice, BLM-induced	5 × 10^5^	IV	2 and 7 days a.t.	Yes	↓ Proliferation and differentiation of MLFs; ↓ apoptosis of MLE-12 cells	Chen et al., 2020

*PF, pulmonary fibrosis; BLM, bleomycin; IV, intravenous; IT, intratracheal; a.t., after treatment; BM, bone marrow; AD, adipose tissue; WJ, Wharton’s jelly; CC16, clara cell secretory protein; KGF, keratinocyte growth factor; COL1A1, type I collagen; CTGF, connective tissue growth factor; TGF-β, tumor growth factor-β; IL-6, interleukin-6; TNF-α, tumor necrosis factor alpha; IP-10, IFN-γ-induced protein 10; MMP-9, matrix metallopeptidase-9; TLR-4, toll-like receptor-4; EMT, epithelial-mesenchymal transition; MLFs, primary mouse lung fibroblasts; MLE-12, murine lung epithelia-12.*

Notably, there is also remarkable heterogeneity among distinct subpopulations of MSCs when viewed at single cell resolution, even from a single origin. Therefore, using the right cells from the right source in the appropriate disease model is likely to impact preclinical efficacy outcomes, and a method to ensure the homogeneity of MSCs is another challenge for the application of MSCs as a commercial drug. In a recent publication (Wu et al., 2020), clinical-grade human embryonic stem cell (hESC)-derived immune and stromal regulatory cells (IMRCs) produced under the requirements of good manufacturing practices (GMP) were reported by Wu et al. IMRCs avoid the ethical controversy of embryonic stem cells while maintaining relatively strong proliferative activity. The expression levels of immunomodulatory and anti-fibrosis genes were found to be higher than those of UC-MSCs. IMRCs derived from hESCs have the advantages of higher homogeneity and immunomodulatory ability and are thus worthy of further investigation.

#### Donor Factors

The therapeutic effect of MSCs is not only affected by tissue source but also by additional donor factors. Whether to use allogeneic or autologous MSCs is the first factor to be considered. Although MSCs from allogeneic donors could be removed faster than autologous ones (Ankrum et al., 2014), the “hit and run” mechanism of MSC function implies that longer residence time might not be crucial. Rodent models of acute lung injury have demonstrated that allogeneic transplantation of BM-MSCs is more effective than autologous transplantation of BM-MSCs, which could be associated with the limited auto-immunoregulatory ability of autologous MSCs (Antebi et al., 2018). Data from clinical trials support these findings. The results of the POSEIDON trial demonstrated that allogeneic MSCs were superior to autologous MSCs for treating patients with decompensated heart failure (Hare et al., 2017).

Further, intrinsic donor factors such as age, sex, and health status might affect the function of transplanted MSCs (Siegel et al., 2013; Brown et al., 2019). The number, proliferation, and differentiation potential of MSCs decrease with aging (Yang, 2018) or obesity (Baptista et al., 2015). Decreased superoxide dismutase activity has been detected in senescent MSCs, suggesting their weak antioxidant ability (Choudhery et al., 2014). Similarly, the anti-fibrotic effects of MSCs derived from aged donors are limited. Tashiro et al. (2015) investigated the difference between intratracheal infusion of AD-MSCs from young and old male mice after the administration of BLM. They observed that the lung tissue of BLM-treated mice receiving young donor AD-MSCs exhibited a decrease in fibrosis, inflammatory markers, oxidative stress, and apoptosis; in contrast, fibrosis and related markers were not decreased after treatment with aged donor AD-MSCs. Another study reported decreased cytokine and chemokine receptor expression in BM-MSCs from aged mice, which reduced the viability of these cells migrating to the injury site and attenuated their protective effects on lung tissue (Bustos et al., 2014). Cell therapy for pulmonary fibrosis is age-dependent. As such, donor age should be considered carefully in cell therapy for IPF patients, as transplantation results can vary according to the age of the cell donor. Donor comorbidities might also influence the potency of MSCs. BM-MSCs from patients with IPF exhibited signs of senescence and did not prevent BLM-induced pulmonary fibrosis in mice (Cárdenes et al., 2018). Given that IPF patients are geriatric, the efficacy of autologous transplantation may be limited. Therefore, allogeneic transplantation might be more recommended, and MSCs from healthy young donors should be preferred.

#### Pretreatment of MSCs

Preclinical animal data indicate that various strategies might enhance the pharmaceutical efficacy of MSCs before infusion, such as changing cell culture methods, adding cytokines or drugs to the culture medium, and modifying MSCs via genetic engineering (Silva et al., 2018; Zhao et al., 2019). MSCs cultured under hypoxic conditions promote the anti-fibrotic activity of radiation-induced and BLM-induced pulmonary fibrosis in murine models. This enhanced potency of MSCs is realized by various mechanisms, including the upregulation of anti-inflammatory, anti-apoptotic, and antioxidant gene expression, as well as changes in proliferation and Akt signaling (Lan et al., 2015; Li et al., 2017a). KGF is a key growth factor involved in the repair of lung epithelial damage. Pretreatment of MSCs with KGF increases their ability to prevent hyperoxia-induced pulmonary fibrosis, which could be underscored by the increased homing of MSCs to fibrotic lung tissue (Yao et al., 2013). Pretreatment with oncostatin M (OSM) prolongs the survival time of transplanted MSCs, upregulates HGF secretion, and enhances the effectiveness of transplantation in the treatment of pulmonary fibrosis (Lan et al., 2017). Pretreatment of MSCs with the diabetes drug pioglitazone *in vitro* increases the expression of VEGF (Hong et al., 2016). Further, MSCs preincubated with the antioxidant N-acetylcysteine exhibit increased intracellular glutathione levels and antioxidant capacity (Wang et al., 2013).

In addition to the preconditioning of MSCs, genetic engineering and stem cell biology to treat diseases has significant therapeutic potential (Min et al., 2015; Huleihel et al., 2017; Liu et al., 2018). In a rat model of silicon dioxide-induced pulmonary fibrosis, allogeneic BM-MSCs modified with BMP-7 were found to inhibit pulmonary fibrosis by reducing epithelial-mesenchymal transition. Compared to that with unmodified BM-MSCs, modified BM-MCSs exhibit enhanced anti-fibrotic capacity (Yang et al., 2016; Li et al., 2017c). The transduction of MSCs with IL-10 or HGF prevents serious side effects caused by the application of MSCs, which might be related to the excessive release of TGF-β from these cells (Islam et al., 2019). Preconditioning strategies and genetic modifications to improve MSC potency in animal models of pulmonary fibrosis are demonstrated in Table 2.

**TABLE 2:** Preconditioning strategies to improve MSC potency in animal models of pulmonary fibrosis.

Preconditioning strategy	Cell source	PF model	Efficacy results	References
Hypoxic environment	BM	Mice, BLM-induced	↑ MSC survival rate ↓ IL-6 and IL-1β mRNA expression/protein levels ↓Lung injury score; collagen deposition Improvement of pulmonary respiratory functions	Lan et al., 2015
KGF	BM	Neonatal rat, hyperoxia-induced	↓ Hydroxyproline in lung homogenates ↑ SHH signaling pathway ↓Lung injury histopathological index	Yao et al., 2013
OSM	BM	Mice, BLM-induced	↑ Survival rates ↓Total cell counts and neutrophil counts in BALF ↓Lung injury score, collagen deposition, ↑ HGF protein levels ↓ TGF-β, CTGF, MMP9 and TIMP1 mRNA levels in lung homogenate	Lan et al., 2017
G-CSF	BM	Mice, BLM-induced	↑ CXCR4 expression ↑homing to the lung ↑antifibrotic effects	Zhao et al., 2019
N-acetylcysteine	Embryonic tissues	Mice, BLM-induced	↓ ROS levels, apoptotic lung cells ↓ Lung injury score, collagen deposition ↓ IL-6, TNF-α, and IL-1β protein levels in BALF ↑ Survival rates	Wang et al., 2013
Overexpress BMP-7 gene	BM	Rat, silicosis-induced	↓ Lung injury score; collagen deposition ↓ Hydroxyproline in lung homogenates ↑ SP-C and AQP-5 protein levels in lung homogenate	Li et al., 2017c
Overexpress Decorin gene	UC	Mice, radiation-induced	↓ Apoptotic lung cells ↓ Lung injury score ↓ Chemokines and inflammatory cytokines ↓Tregs	Liu et al., 2018
Overexpress ACE2 gene	UC	Mice, BLM-induced	↓TNF-α, IFN-γ, TGF-β, IL-1, IL-2, IL-6 ↓ Lung injury score; hydroxyproline concentration ↑SOD, GSH, ACE2 and IL-10	Min et al., 2015
Overexpress miRNA let-7d	BM	Mice, BLM-induced	↑ mice weight and survival ↓ Collagen-1 expression ↓ CD45-positive cells	Huleihel et al., 2017

*KGF, keratinocyte growth factor; OSM, oncostatin M; G-CSF, granulocyte colony-stimulating factor; CXCR4, CXC chemokine receptor-4; TIMP1, tissue inhibitor of metalloproteinase 1; BMP7, bone morphogenetic protein-7; UC, umbilical cord; ACE2, angiotensin-converting enzyme 2 gene; DEG, differentially expressed genes.*

In general, preconditioning or genetic modifications can enhance MSC therapeutic potential. However, the adverse effects of pretreatment of MSCs are less well studied. For example, researchers have applied viral-based gene modification strategies because this system is efficient and stable. However, viral transduction is associated with many risks, such as immunogenicity and chromosomal integration risks (Oggu et al., 2017). Therefore, further research is needed to evaluate both the efficacy and safety of pretreated MSCs.

### Intervention Time

The American Thoracic Society advocates for the intratracheal BLM mouse model as the optimal animal model for preclinical testing (Jenkins et al., 2017). The advantage of using BLM in the mouse trachea is the requirement for only a single infusion, enabling the reproduction of fibrotic diseases in a short time. The BLM model can be divided into three stages. The first 3 days after installation is the inflammation triggering stage. Fibroblasts proliferate after 7 days, and the fibrosis model is established 15 days later (Serrano-Mollar, 2018). In the early stage of the mouse inflammation model, stem cell therapy reduces inflammatory factors, activates fibroblasts and collagen deposition promotes, epithelial cell repair, and exerts a significant protective effect on damaged tissues (Li et al., 2017b; Rubio et al., 2018). Early experimental data only support the potential improving effect of MSC administration on fibrotic lung diseases if administered in the early stage of the disease, during active inflammation. However, most patients who present with respiratory symptoms have already developed pulmonary fibrosis to varying degrees. As using MSCs for early intervention is impractical, it is necessary to evaluate the beneficial effect of MSC transplantation on established lung fibrosis. Fortunately, several recent studies have shown that MSCs also have a therapeutic effect on established pulmonary fibrosis models. In a BLM-induced rat pulmonary fibrosis model, BM-MSCs were administered 14 days after BLM injection. MSCs inhibited the expression of TGF-β/Smad3 and reduced the deposition of collagen fibers in lung tissue (Gad et al., 2020). In another study, UC-MSCs were administered 21 days after the intratracheal instillation of BLM. UC-MSCs activated the host macrophages to synthesize MMP-9 and degrade existing collagen (Chu et al., 2019). In a silica-induced fibrosis model, the effects of late MSC transplantation were also promising (Zhang et al., 2018). These experimental data indicated that although early MSC intervention might be more effective, MSCs application at a later time could be also beneficial, supporting such strategies to treat pulmonary fibrosis.

### Interventional Approach

Understanding the biodistribution of cells in stem cell therapy is an important step in translating preclinical findings into clinical settings. Two methods are commonly used to introduce MSCs into target tissues, systemic and local delivery (Kean et al., 2013; Cardenes et al., 2019). In most preclinical studies, MSCs are delivered via the intravenous (IV) route in pulmonary fibrosis models (Reddy et al., 2016; Chen et al., 2018; Moroncini et al., 2018; Ni et al., 2018; Wu et al., 2020). Intravascular delivery is based on the theory that (1) the delivered MSCs will receive and respond to the injury signals released by damaged airway tissue, and these stimuli will induce MSCs to home from the blood vessels to the injury site (Nitzsche et al., 2017; Abdel Halim et al., 2020) and (2) that MSCs will interact with distant cells via endocrine pathways to promote regeneration (Khatri et al., 2018; Willis et al., 2018). The preferred choice of the intravenous route might be due to easy and practical procedures, which allow for repeated transplantation.

In contrast, local administration is advantageous in that it prolongs the cell half-life, improves utilization, and reduces side effects on other organs (off-target effects). In pulmonary diseases, intratracheal (IT) route is also used, and showed effects in IPF models (Chu et al., 2019; Ji et al., 2020). In addition to traditional IT injection, another IT delivery method is aerosol technology, which is widely used to deliver pharmaceutically active substances to the lungs (Ehrmann et al., 2020). Kim et al. (2016) reported that spraying human amniotic MSCs onto substrates of different stiffnesses enabled cell survival. In preclinical studies of acute and chronic lung injury in rabbits, Kardia et al. (2018) observed that aerosol-based cell delivery was a feasible technique to deliver cells directly to the lungs and to evenly distribute nebulized solutions containing cells. In addition to stem cells, aerosol technology is an ideal tool for delivering cell-derived biologics, particularly MSCs-EVs (McCarthy et al., 2020).

Regarding the effect of different approaches on the therapeutic effect of MSCs, Cardenes et al. (2019) compared the IT or IV routes of bone marrow-derived multipotent adult progenitor cell administration in an ovine model of acute respiratory distress syndrome (ARDS). PET/CT images revealed that IT-administered cells remained at the administration site and no changes were observed within 5 h, whereas intravenously administered cells exhibited a wide biological distribution, with the highest cell concentration in the lungs. Meanwhile, the biological distribution of cells varies depending on the method of administration, and both routes of administration led to comparable therapeutic effects. In emphysema models, different routes of delivery MSCs showed different effects. IV injection of MSCs could reduce the deterioration in lung function, but no protective effect was observed in the IT route. IV injection of MSCs creates a systemic anti-inflammatory response via the systemic circulation on the endothelial surface of the pulmonary vasculature. Thus, the anti-inflammatory milieu on the endothelial side produced by MSCs injected via the vein could decrease the vascular inflammation shown to be present in the elastase-induced emphysema model (Tibboel et al., 2014). It is worth noting that the effect of different MSCs intervention approaches on lung diseases may be different from other diseases. As in perianal fistulizing Crohn’s disease (PFCD), a meta-analysis published in 2015 showed that local injection of MSCs into fistula lesions has considerable therapeutic benefit, which is more significant than systematic administration (Dave et al., 2015). It is speculated that MSCs maintain local anti-inflammatory effects by sending paracrine signals to neighboring cells. Whereas the systemic application of MSCs may not allow a sufficient number of MSCs to migrate to the inflammatory site, and the dosage could be limited by the potential systematic side-effect (Gallo et al., 2020). This difference of therapeutic effect may be due to the different characteristics of different diseases. In pulmonary fibrosis, the focus is often diffusely distributed, rather than concentrated in a certain point. Therefore, the effect of tracheal administration and local administration in PFCD is not the same, which may be the reason why IV and IT routes are both effective in pulmonary fibrosis. Further, the intraperitoneal injection of MSCs was reported to have therapeutic effects on pulmonary fibrosis induced by repeated BLM treatments (Zhang et al., 2012). This suggests that the direct contact of stem cells with lung tissue might be unnecessary, or different mechanisms may play a role in different interventional approaches, which is worthy of further study in details. The effects of different delivery methods on MSC efficacy are shown in Table 3.

**TABLE 3:** Different delivery routes of MSCs in animal models of pulmonary disease.

Delivery	Cell Source	Animal models	Dose	Efficacy Results	References
Intraperitoneal	BM	BPD	1 × 10^5^	Improves survival rate Prevents pulmonary fibrosis	Zhang et al., 2012
Nebulization	UC-CM/BM-CM	ARDS	10 mL	Anti-bacterial ability remained after nebulization	McCarthy et al., 2020
IV	UC	Lung fibrosis	2.5 × 10^5^	Anti-fibrotic activity	Moroncini et al., 2018
IT	Amniotic	Lung fibrosis	1 × 10^6^	Inhibit B cell activation	Cargnoni et al., 2020
IV vs. IT	BM	Pulmonary emphysema	IV: 1 × 10^5^ IT: 5 × 10^5^	IV is better than IT	Tibboel et al., 2014
IV vs. IT	BM	ARDS	IV: 10 × 10^6^ cells/kg IT: 1 × 10^6^ cells/kg	IV is similar to IT	Cardenes et al., 2019

*CM, conditioned medium; BPD, bronchopulmonary dysplasia.*

### Infusion Dose and Speed

Similar to drugs, the therapeutic effects of MSCs are dose-dependent (Jun et al., 2011; Schlosser et al., 2019). Therefore, the minimum effective dose and optimal dose range of cell preparations need to be further specified before their widespread application. Early dosage is difficult to evaluate from traditional preclinical pharmacokinetics and pharmacodynamics, but previous clinical experience might provide some guidance. An analysis of clinical trials involving MSCs from 2004 to 2018 by Kabat et al. (2020) revealed that the minimum effective dose (MED) was 70–190 × 10^6^ MSCs per patient among trials with positive results reported. Only four of the trials reported data on dose-effect relationships and indicated that the MED range was narrow (between 100 and 150 × 10^6^ MSCs per patient). The clinical effects are insufficient if the number of cells is too low, but excessive cells could increase the risk of pulmonary embolism (Janowski et al., 2013; Liu et al., 2017), leading to increased mortality. Anticoagulation therapy with heparin before the application of high-dose MSCs might prevent serious adverse events (Liao et al., 2017). In addition, infusion speed is a key factor that affects the safety of cell therapy (Janowski et al., 2013). In experiments on rats, the risk of stroke was found to be significantly increased when the infusion speed exceeded 1 mL/min, whereas a lower speed (0.2 mL/min) was relatively safe.

### Interval and Frequency of Intervention

The majority of studies have focused on the safety of stem cell transplantation and only employed MSCs once. However, following preliminary safety tests, it is necessary to address the issues of the administration interval and frequency. Technological advances in *in vivo* tracing have enabled superior tracking of the biodistribution and clearance of MSCs in the body to optimize delivery strategies. It has been reported that the time that MSCs remain in lung tissue varies from 7 days to 4 months, depending on the detection method and disease model used (Leibacher and Henschler, 2016; Abdel Halim et al., 2020). Poggio et al. (2018) compared the intervention strategies of MSCs administered once and twice with a second dose weekly and reported that repeated administration had stronger immunosuppressive effects on T cells, and mainly CD8^+^ T cells. In a murine model of BLM-induced pulmonary fibrosis, repeated administration of MSCs three times every 3 days had comparable antifibrotic effects to the continuous administration of pirfenidone (Reddy et al., 2016). Similar results were obtained in rat models of radiation-induced pulmonary fibrosis (Zhang et al., 2019). Considering the short half-life of donor MSCs *in vivo*, multiple transplantation times is a promising solution.

## Efficacy and Safety of Clinical Trials With Different Parameters

Preclinical studies in rodents have achieved unprecedented positive results, which will encourage the translation of MSC therapy into clinical trials. Currently, multiple phase I clinical trials have been conducted to ensure the safety of MSC treatment (Table 4). A phase Ib study of the intrabronchial administration of autologous AD-MSCs in IPF patients reported acceptable safety and improved parameters of life quality (Tzouvelekis et al., 2013). The recently published longitudinal results of this study further supported the safety of cellular therapy. In 14 patients, the 2 year survival rate was 100% after the first dose, with progression-free survival of up to 26 months without tumors (Ntolios et al., 2018). In addition, studies on the intravenous injection of placenta-derived MSCs (Chambers et al., 2014) and BM-MSCs (Liu et al., 2015; Glassberg et al., 2017; Averyanov et al., 2020) have revealed that it is safe for IPF patients to receive up to 2 × 10^8^ cells without experiencing hemodynamic abnormalities, which relieves the concern that the application of MSCs to a damaged vascular system might increase the risk of pulmonary embolism. These clinical trials have paved the way for recruiting more patients and further studies on dosage.

**TABLE 4:** Summary of current phase I clinical trials on ILD treatment with MSCs.

Trial number	Disease	Nationality	Number of patients	Cell population	Number of cells	Administration route	Times of administration	Follow-up period (month)	References
NCT02013700	IPF	American	9	Allogenic BM-derived	20 × 10^6^ 100 × 10^6^ 200 × 10^6^	IV	Single	60	Glassberg et al., 2017
NCT01977131	Silicosis	Chinese	4	Autologous BM-derived/HGF	2 × 10^6^/kg	IV	Once a week, repeat three times	12	Liu et al., 2015
NCT01385644	IPF	Australian	8	Allogenic, PL-derived	1 × 10^6^ 2 × 10^6^	IV	Single	6	Chambers et al., 2014
NCT02594839	IPF	Russian	20	Allogenic BM-derived	200 × 10^6^	IV	Twice a week, repeated 12 weeks apart, 4 times in total	12	Averyanov et al., 2020
NCT N/A	IPF	Greek	14	Autologous AD-derived	0.5 × 10^6^/kg	IT	Once a month, repeat three times	12	Tzouvelekis et al., 2013
NCT02135380	IPF	Indian	60	Autologous AD-derived	2 × 10^6^/kg	IV	Once a week, repeat three times	9	Unpublished
NCT02277145	RP	Chinese	10	Allogenic UC-derived	1 × 10^6^/kg	IT	Single	6	Unpublished
NCT01919827	IPF	Spanish	17	Autologous BM-derived	N/A	IT	Single	12	Unpublished

*N/A, not available; RP, radiation pneumonia; IPF, idiopathic pulmonary fibrosis; BM, bone marrow; PL, placenta; AD, adipose; UC, umbilical cord; IT, intratracheal; IV, intravenous. Data are collected from clinicaltrials.gov as of November 2020.*

The results of the AETHER trial (Glassberg et al., 2017) demonstrated the safety of intravenous infusion of allogeneic BM-MSCs in IPF patients. No serious adverse events were observed during the 60-week follow-up period, and the most common adverse reactions were bronchitis and cold. The predicted value of FVC decreased by 3.0% on average, and the predicted value of carbon monoxide diffusing capacity (DLCO) decreased by an average of 5.4%, which was below the internationally recognized threshold of disease progression. Comparisons of patients receiving doses of 2 × 10^7^ and those receiving 10 × 10^7^ MSCs revealed that the HRCT fibrosis score of patients in the high-dose group progressed more slowly, with a slower rate of DLCO decrease and improved treatment effects (Fishman et al., 2019). These results suggest that further large-scale dose escalation studies should be conducted to determine the optimal dose of MSCs.

In clinical trials, the strategy of MSC pretreatment has been applied to treat pulmonary fibrosis. For example, 2 × 10^6^ cells/kg of autologous BM-MSCs were infused intravenously into four silicosis patients twice per week for three consecutive weeks. These MSCs were transfected with *HGF cDNA* vectors before infusion (Liu et al., 2015). Patient clinical symptoms such as cough and chest tightness were alleviated after 6 months, with a significant improvement in lung function, serum IgG levels, and circulating T-lymphocyte levels, thereby confirming the safety of premodified MSCs.

These trials used autologous or allogeneic BM-MSCs, AD-MSCs, PL-MSCs, and UC-MSCs, different doses ranging from 1 to 200 × 10^6^ MSCs, and different routes of administration thus making comparisons difficult. To date, the majority of clinical trials are in the early stages of evaluating the safety, feasibility, tolerance, and efficacy of cell therapy. The limitations of these studies include small sample sizes and a lack of randomization and/or placebo controls. Cell therapy requires the consideration of further optimization of stem cell type, *in vitro* modification, intervention schemes, routes of administration, and suitable dosage. Nevertheless, these reports suggest that stem cell transplantation in IPF is initially safe and has some benefits, and standardized protocols might encourage scientists to explore the potential of MSC therapy for IPF patients.

## Other Factors Potentially Affecting the Safety of MSC Therapy

Data from preclinical animal experiments are encouraging, and serious adverse events have not been reported in clinical trials, highlighting the safety and feasibility of MSC transplantation. Nonetheless, clinical trials must be conducted with caution. Since TGF-β has been validated as a causative factor of fibrosis (Kim et al., 2018), there are concerns that its secretion by MSCs could promote abnormal fibroblast populations in the context of fibrosis and immune system abnormalities (Salazar et al., 2009). Correcting the microenvironment prior to the use of MSCs will enhance the benefits and eliminate the side effects of fibrosis (Islam et al., 2019). Another concern is the possibility of tumor formation after transplantation (Ben-David et al., 2011). However, Sensebé et al. (2012) refuted this view and proposed that human adult stem cells and MSCs cultured *in vitro* have strong genetic stability. Systematic evaluation and meta-analyses of clinical trials in stem cell therapy for various respiratory diseases have not reported serious adverse events after MSC transplantation such as the formation of transplant-related tumors (Zhao et al., 2017). The quality control of MSCs is a critical step before the clinical application of regenerative therapy. MSCs used in clinical settings should conform with the principles of GMP (Torre et al., 2015; Samsonraj et al., 2017). Further refinements are required to ensure the stability of cell preparations, as factors such as donor heterogeneity, *in vitro* amplification, immunogenicity, and cryopreservation might influence the therapeutic efficiency and safety of MSCs (François et al., 2012; Wang and Han, 2019).

## Conclusion

With the developments in regenerative medicine technology, stem cell therapy has been tested for safety and efficacy in various lung diseases in the last decade. Based on the results of IPF preclinical animal models and clinical trials, as well as the urgent need for new treatments, well-designed and optimized clinical trials of MSC therapy for IPF patients are warranted. Safety should be the primary concern in early clinical trials, followed by clinical and biological efficacy. Significant parameters that will influence the effectiveness of MSCs must be considered before conducting large-scale trials, including cell source, the donor and recipient, route, dose and time of administration, and pretreatment of MSCs, to maximize their therapeutic efficacy while minimizing potential side effect.

## Author Contributions

SY and PL conceived of the idea, performed the literature search, collected the data, and drafted the entire article. YJ performed a review of clinical trials. ZW, HD, and CW revised the manuscript. All authors contributed to the article and approved the final version of the manuscript.

## Conflict of Interest

The authors declare that the research was conducted in the absence of any commercial or financial relationships that could be construed as a potential conflict of interest.
